# Median nerve conduction impairment in patients with diabetes and its impact on patients’ perception of health condition: a quantitative study

**DOI:** 10.1186/1758-5996-5-16

**Published:** 2013-03-25

**Authors:** Jolanta Lewko, Barbara Polityńska, Jan Kochanowicz, Wiesław Zarzycki, Zenon Mariak, Maria Górska, Elżbieta Krajewska-Kułak

**Affiliations:** 1 Department of Integrated Medical Care, Medical University of Bialystok, M. Skłodowskiej-Curie str. 7A, Bialystok, 15-096, Poland; 2 Department of Philosophy and Human Psychology, Medical University of Bialystok, Szpitalna str. 37, Białystok, 15-295, Poland; 3 Department of Invasive Neurology, Medical University of Bialystok, Marii Skłodowskiej-Curie str. 24A, Białystok, 15-276, Poland; 4 Department of Neurosurgery, Medical University of Bialystok, Marii Skłodowskiej-Curie str. 24A, Białystok, 15-276, Poland; 5 Department of Endocrinology, Diabetes and Internal Medicine, Medical, University of Bialystok, Marii Skłodowskiej-Curie str. 24A, Białystok, 15-276, Poland

**Keywords:** Median nerve, Diabetic neuropathy, Quality of life

## Abstract

**Introduction:**

Impaired mobility and compromised manual dexterity leading to difficulties with the activities of daily living (ADL) are an inherent part of the clinical picture in diabetes. Hand function in diabetes is influenced by a variety of pathologies: the median nerve, the most important nerve of the hand, can suffer from metabolic disturbances, ischemia and/or entrapment neuropathies. The resulting deterioration in functional capacity is likely to have significant consequences for the ability to perform ADL, influencing adjustment to diabetes and affecting quality of life. The aim of the present study was to examine the influence of hand function as measured by median motor nerve conduction on quality of life, taking into account various aspects of functioning in patients with diabetes, including activities of daily living, psychological status and acceptance of illness.

**Patients and methods:**

Seventy one hospital patients with diabetes participated in the study. Electrophysiological recordings of conductance in the median nerve were obtained for both hands and the relationship between hand function and functional status (BI), depression and anxiety (HADS), adjustment to illness (AIS) and their effect on quality of life (SF-36v2 and QLI) was studied.

**Results:**

Damage to the median nerve of the left hand was associated with significant differences in functioning in the physical, but not the mental component of the SF-36v2, p = 0.03 and in functional status (p = 0.006). QOL was associated with depression, patient age, acceptance of illness, functional ability and to a small, but significant extent with median nerve damage to the right hand on the measure of conduction velocities (R2 =0.726).

**Conclusions:**

Nerve conductance studies demonstrated a small, but significant effect of hand function on quality of life. Impairment of the median nerve in the left hand was associated with functional difficulties in the activities of daily living and a diminished quality of life in the area of physical functioning. No dependencies of this kind were found for the right hand, which may reflect the greater compensatory capacity of the right hand resulting from improved efficiency due to practice.

## Introduction

It is well established that diabetes is associated with various complications, among the most common of which are the peripheral neuropathies, often leading to functional impairments affecting mobility and the activities of daily living (ADL) [1,2]. The peripheral neuropathies mostly affect the lower limbs which, as a result, have been the focus of considerable research [3-7]. However, they may eventually also involve the hands [8,9] and diminished hand strength has been associated with functional disability in diabetes [10].

Hand function in diabetes is influenced by a variety of pathologies, the most common presentations being carpal tunnel syndrome, trigger finger, Dupuytren’s contracture and limited joint mobility. The median nerve of the hand, whose integrity is vital to normal hand function, can suffer from metabolic disturbances, ischemia and/or entrapment neuropathies. Nevertheless pathology in this nerve often goes unrecognised: the signs of carpal tunnel syndrome (CTS) are found in 20-30% of diabetic patients on electrophysiological examination [11], whereas clinical signs are recognised in only 5.8% of patients [12]. Moreover, the syndrome occurs three times more frequently in diabetes than in the general population [11]. Equally, diabetes can affect the median nerve as a mononeuropathy or as part of a systemic polyneuropathy, both conditions being associated with widespread nerve damage, not confined to the carpal region [13]. Median nerve dysfunction would be expected to cause impaired hand function in the form of decreased muscle strength, pain and impaired sensation and in consequence to lead to an increased risk of burns and hand ulceration [14].

It is reasonable to expect, that even mildly compromised hand function in diabetes, which restricts the patient’s pre-morbid dexterity and limits activities requiring fine manual skills, might be reflected in feelings of deteriorated functional capacity, resulting in lower subjective ratings of illness acceptance and mood and affecting perceptions of quality of life. Very few studies have examined the relationship between hand impairments, functional ability and quality of life in patients with diabetes: Savas et al. [10] obtained electrophysiological measurements for the sural, median and peroneal nerves, but these parameters did not correlate with their measure of functional disability for the hands; and Padua et al. assessed nerve conductance in the sural, peroneal, and ulnar nerve, but not in the median nerve, in relation to quality of life [15]. However, most studies have either addressed quality of life in diabetes more generally or those that have examined hand function, for example in relation to touch and temperature sensation [2], restricted hand movement, Dupuytren’s contracture [16], or the symptoms of carpal tunnel syndrome [17] have not attempted to evaluate these in terms of quality of life.

Diabetes-related morbidity [18] and the peripheral neuropathies in particular, [19-21] are known to be associated with poorer quality of life as reported by patients with diabetes. Quality of life is regarded as an important health outcome indicator in diabetes, alongside survival time and the successful prevention and management of complications, as it takes account of the effect of the illness on various aspects of the patient’s life as judged by the patient him/herself and provides important information in addition to the standard medical evaluation [22,23]. As these evaluations are made in the context of a long-term illness, the term ‘health related quality of life’ (HRQoL) is used. Since there is no universally accepted ‘gold standard’ instrument that might provide a holistic evaluation of patients with diabetes, an optimal solution might be to combine both generic and diabetes-specific instruments in the assessment of patients, and this was the path taken in the present study. Furthermore, despite the multidimensionality of instruments used to measure quality of life there is evidence that they are measuring dimensions that are distinct from psychopathology, and thus instruments to measure depression and anxiety as well as illness acceptance were also included, as it seemed likely that these might contribute to quality of life in diabetes.

Thus, the aim of the present study was to investigate the relationship between hand function as measured by median nerve motor conduction and quality of life in diabetes, taking into account a range of different variables, such as performance in the activities of daily living, reported levels of illness acceptance, anxiety and depression. In the first instance, the relationship between demographic (e.g. age and gender) and clinical variables (e.g. duration and severity of the illness) and impairment in median nerve motor conduction was examined in order to determine any effects of these variables on hand function. Potential differences between right and left hands were also examined.

## Methodology

### Subjects

Seventy one patients admitted consecutively to the Department of Endocrinology, Diabetology and Internal Medicine of the Medical University Hospital in Białystok, Poland participated in the study. Twenty one of the patients had a diagnosis of type 1 and 50, type 2 diabetes and all were right handed. Their mean age was 54.6 ± 14.3 years, (range 18–80 years); for the 37 women participating in the study, it was 56.1 ± 14.4 years and for the 34 men, 53.0 ± 14.3 years, with a mean illness duration of 18.2 years for women and 9.9 years for men. Levels of HbA1c were used as an index of glycemic control, normal values of which should be ≤ 7% [24].

### Inclusion/exclusion criteria

Patients with both type 1 and type 2 diabetes were included in the study; their selection was based on the following criteria: age below 80 years, satisfactory glycaemic control with a blood glucose level of 90–140 mg% on the day of examination and on the preceding day. Patients with the symptoms of Dupuytren’s disease, CTS and neurological conditions other than diabetic neuropathy and those who were confined to bed or fitted with a pacemaker were excluded from the study.

### Ethical approval

Ethical permission for the study was obtained from the Bioethical Committee of the Medical University of Bialystok (R-I-002/115/2009). All patients were fully appraised with the study protocols and gave their informed consent.

### Procedure

Motor nerve conduction studies of the median nerve (distal motor latency-DML and conduction velocity -CV) were performed according to standard techniques by means of Keypoint equipment for all individuals [25]. All the tests were carried out by a nurse trained in the procedures, at an ambient room temperature of 22-25°C. A bipolar electrode (9013 L0361) was used to stimulate the median nerve and median motor onset latency was recorded at the level of the elbow and wrist. Median nerve neuropathy was diagnosed at distal latencies greater than 3.8 ms and conduction velocities below 50 m/s in accordance with normal reference values established in our laboratory; these values were taken as the threshold for impairment on the electrophysiological measures recorded [26].

### Measures

Health related quality of life (HRQoL) was assessed using the SF-36v2 [27] which provides summary scores relating to the physical (PCS) and mental (MSC) components of HRQoL, and is a generic measure in which higher scores indicate better quality of life, and the Quality of Life Index (QLI) for Diabetes Version III, [28,29], which is a disease-specific measure of quality of life in diabetes, with scores ranging from 0–30, higher scores reflecting improved quality of life. Both instruments were used in order to sample as wide a range of domains of quality of life as possible, in keeping with the criteria outlined in the introduction. Since hand dysfunction might be expected to be reflected in functional abilities, a measure of performance on the activities of daily living (ADL) was obtained with the aid of the Barthel Index (BI): the scale is scored from 0–100, higher scores being indicative of better functional performance [29,30]. Measures of anxiety and depression were obtained using the Hospital Anxiety and Depression Scale (HADS) in which scores for each of the sub-scales (anxiety or depression) range from 0–21, higher scores indicating greater psychopathology [29], and the degree of acceptance of illness was evaluated using the Acceptance of Illness Scale (AIS) [31], which consists of statements describing the negative consequences of having poor health status, scale scores range from 0–40, higher scores being associated with better illness acceptance. These two measures were included, as being potentially likely to influence quality of life in diabetes. All of the instruments have been validated for use in clinical populations.

### Analysis

Data analyses were carried out using the SPSS 17.0 statistical package. Nonparametric tests were used because of the non-normative or categorical nature of the data. Comparisons between groups were made using the Odds Ratio (OR), the Chi-square test for categorical data and the Mann–Whitney test in the case of continuous variables. All tests were two tailed. The significance level accepted was p < 0.05. Spearman rank correlations and step-wise linear multiple regression analyses were used to establish relationships in the data. Predictor variables for the regression model included the CV for both hands, the demographic and clinical variables found to discriminate between groups (gender and HbAIC) and age of the patients, which although not significantly different between groups, showed some differences (see Table 1). Other predictor variables of special interest included: functional ability, as measured on the Barthel (BI), the degree of illness acceptance (AIS) and the anxiety and depression measures obtained on the HADS. The two dependent variables consisted of the generic (SF36) and the specific (QLI) measures of quality of life.

**Table 1:** Clinical characteristics of the diabetic patients examined according to impaired versus unimpaired DML and CV of the median nerve for both hands

	**Distal motor latency (mean ± SD ms) (DML)**						**Conduction velocities (mean ± SD m/s) (CV)**					
	**Left hand**			**Right hand**			**Left hand**			**Right hand**		
	**Unimpaired**	**Impaired**		**Unimpaired**	**Impaired**		**Unimpaired**	**Impaired**		**Unimpaired**	**Impaired**	***P value***
	**N = 30**	**N = 41**	***P value***	**N = 22**	**N = 49**	***P value***	**N = 48**	**N = 23**	***P value***	**N = 41**	**N = 30**	
	**Mean = 3.4 ±0.2 ms**	**Mean = 4.6 ± 0.9 ms**		**Mean = 3.4 ± 0.3 ms**	**Mean = 4.9 ± 1.2 ms**		**Mean = 54.5 ± 3.5 m/s**	**Mean = 46.1 ± 2.8 m/s**		**Mean = 54.1 ± 3.4 m/s**	**Mean = 45.8 ± 2.7 m/s**	
Age (years)	51.3 ± 16.8	56.9 ± 12.1	ns	52.3 ± 16.4	55.7 ± 13.3	ns	54.7 ± 15.4	54.4 ± 12.3	ns	52.8 ± 14.9	57.0 ± 13.4	ns
Gender												ns
Women N(%)	17(56.7%)	20(48.8%)	ns	10(45.5%)	27(55.1%)	ns	29(60.4%)	8(34.8%)	0.002	22(53.7%)	15(50%)	
Men N(%)	13(43.3%)	21(51.2%)		12(54.5%)	22(44.9%)		19(39.6%)	15(65.2%)		19(46.3%)	15(50%)	
Type of diabetes												ns
Type I N(%)	12(40%)	9(21.9%)	ns	7(31.8%)	14(28.6%)	ns	14(29.2%)	7(30.4%)	ns	12(29.3)	9(30%)	
Type II N(%)	18(60%)	32(78.1%)		15(68.2%)	35(71.4%)		34(70.8%)	16(69.6%)		29(70.7%)	21(70%)	
Duration of diabetes (yrs)	13.6 ± 9.4	14.5 ± 11.1	0.03	12.5 ± 8.6	14.8 ± 11.1	ns	13.8 ± 11.1	14.7 ± 8.7	ns	12.2 ± 8.2	16.6 ± 12.3	ns
Hb A1C (%)	8.4 ± 2.9	9.3 ± 1.9	ns	9.2 ± 2.2	8.8 ± 1.9	ns	8.4 ± 1.7	9.8 ± 2.1	0.004	8.5 ± 1.8	9.4 ± 2.1	0.05
BMI (kg/m^2^)	30.5 ± 15.2	27.9 ± 4.9	ns	31.3 ± 17.2	28.0 ± 5.1	ns	29.3 ± 8.4	28.5 ± 6.6	ns	30.5 ± 13.1	27.1 ± 4.9	ns

Data are presented as means ± SD or%; group comparisons (impaired vs. unimpaired) were made using the odds ratio test for non-normally distributed continuous variables and the chi-square test for categorical variables; BMI = body mass index, Hb A1C = c-fraction of glycosylated hemoglobin. *P* values are expressed if they are <0.05.

## Results

The clinical characteristics of the patients studied are presented in Table 1; the sample is divided into those with impaired versus unimpaired conduction in the median nerve in both hands. The mean values (±SD) for the impaired versus unimpaired distal motor latencies (DML) and conduction velocities (CV) are presented. According to the measure of DML, impairment of the median nerve in the right hand occurred in 49 patients and in the left hand in 41 patients, whilst on the basis of CV, impairment was found for 30 patients in the right hand and 23 in the left. Impairment of function was found to occur with similar frequencies in both right and left hands (DML: OR = 1.63; p = 0.22 and CV: OR = 1.53; p = 0.30). The differences in classification between DML and CV may reflect the different sensitivity of the measures to damage in the median nerve; DML is likely to be a measure of early impairment, as distal parts of the nerve are the first to show neuropathic damage.

Within the sample studied, no significant differences were found between impaired and unimpaired groups on either of the median nerve measures (DML or CV) for either hand with respect to the patient’s age, BMI and type and duration of diabetes. The only exception to this was in relation to the left hand, where longer illness duration was associated with DML impairment (see Table 1); a similar trend was also seen for the right hand for DML, and for both hands with respect to CV, but these comparisons did not reach statistical significance. Statistically significant differences were found with respect to CV, for men but not for women, and these differences existed only for the left hand (OR = 0.04; p = 0.002). HbA1c values for the patient sample were high, indicating poor overall glycemic control. Statistically significant differences occurred in the left hand in relation to the levels of HbA1c between the patients with impaired and non-impaired DML (OR = 1.51; p = 0.03) and CV (OR = 1.85; p = 0.004) for the median nerve. For the right hand, the difference between impaired and unimpaired groups occurred only for CV (OR = 1.36; p = 0.05). Thus significant differences between impaired and unimpaired groups were established mainly on measures of CV and with respect to the left hand, in relation to male patients and to levels of HbA1c.

Table 2 and Table 3 show the results of the functional assessment in relation to hand dysfunctions as measured by DML and CV. No differences between the groups were found for ratings of acceptance of illness (AIS) and depression or anxiety scores (HADS) using the Mann–Whitney test. However, significant group differences occurred with respect to the measure of activities of daily living (BI) for DML (p = 0.005) (see Table 2) and CV (p = 0.03) (see Table 3) for the left hand, but not for the right. Damage to the median nerve of the left hand (increased DML) was associated with statistically significant differences in the functioning of the patient in the physical component (PCS) of the SF36v2 (p = 0.01), but not in the mental component (MCS). Left hand DML were also associated with differences in the following subscales: physical functioning (PF; p = 0.05) and physical role functioning (RP; p = 0.03) (Table 2). The CV measure for the left hand discriminated between impaired and unimpaired groups with respect to the general health (GH) subscale (p = 0.03) and bodily pain (BP) subscale (p = 0.03) of the SF36v2 (Table 3). Significant differences were observed between CV impaired and unimpaired groups in relation to QLI for the left hand (p = 0.05) with respect to the health and functioning subscale (HFSUB; p = 0.05), the psychological and spiritual subscale (PSPSUB; p = 0.05) and overall quality of life (QLI-total; p = 0.05). As regards DML, differences between groups were found for the left hand only in relation to the social and economic subscale of the QLI (SOCSUB; p = 0.01). No differences between impaired and unimpaired groups with respect to DML and CV were found for the right hand on any of the functional assessment variables; only deficits in nerve conductance as assessed with CV appeared to distinguish between groups in relation to the family subscale for QLI (FAMSUB; p = 0.03).

**Table 2:** Functional assessment in the physical and psychological domains in relation to impaired versus unimpaired DML of the median nerve for both hands

	**Distal motor latency (mean ± SD ms) (DML)**					
	**Left hand**			**Right hand**		
	**Unimpaired**	**Impaired**	***P value***	**Unimpaired**	**Impaired**	***P value***
	**N = 30**	**N = 41**		**N = 22**	**N = 49**	
	**Mean =3.4 ±0.2 ms**	**Mean = 4.6 ±0.9 ms**		**Mean = 3.4 ± 0.3 ms**	**Mean = 4.9 ± 1.2 ms**	
AIS	31.7 ± 7.9	29.1 ± 8.6	ns	29.1 ± 8.7	30.6 ± 8.2	ns
CI 95%	28.8-34.7	26.3-31.8		25.2-32.9	28.3-33.1	
HADS-D	4.8 ± 4.0	5.6 ± 5.1	ns	5.3 ± 5.2	5.3 ± 4.4	ns
CI 95%	3.34-6.33	4.0-7.2		2.9-7.6	4.0-6.6	
HADS-A	5.6 ± 4.2	5.7 ± 5.3	ns	5.8 ± 4.8	5.6 ± 4.9	ns
CI 95%	4.07-7.19	4.1-7.4		3.7-7.9	4.2-7.1	
BI	99.2 ± 2.9	93.9 ± 10.6	0.005	97.3 ± 6.8	95.6 ± 9.3	ns
CI 95%	98.1-100.3	90.6-97.3		94.2-100.3	92.9-98.3	
SF-36v2						
PF	49.1 ± 9.4	42.9 ± 13.1	0.05	46.9 ± 10.9	44.9 ± 12.5	ns
CI 95%	45.7-52.7	38.8-47.1		42.0-51.8	41.4-48.5	
RP	44.3 ± 12.5	37.8 ± 13.1	0.03	41.1 ± 4.2	40.4 ± 12.8	ns
CI 95%	39.6 = 48.9	33.7-41.9		34.7-47.4	36.7-44.1	
BP	52.5 ± 12.5	46.6 ± 14.5	ns	51.3 ± 12.2	48.2 ± 14.6	ns
CI 95%	47.9 = 57.2	42.1-51.2		45.9-56.7	43.9-52.3	
GH	35.6 ± 12.4	35.3 ± 12.4	ns	37.2 ± 19.9	34.6 ± 11.6	ns
CI 95%	30.9-40.2	31.6-39.1		31.4-42.9	31.3-37.9	
VT	46.3 ± 9.1	45.7 ± 12.4	ns	46.5 ± 9.8	45.7 ± 11.94	ns
CI 95%	42.8-51.6	41.8-49.6		42.2-50.9	42.3-49.1	
SF	47.1 ± 11.8	40.5 ± 15.0	ns	42.7 ± 14.9	43.6 ± 13.8	ns
CI 95%	42.8-51.6	35.7-45.2		36.1-49.3	39.6-47.5	
RE	43.1 ± 13.9	39.6 ± 13.9	ns	42.3 ± 13.9	40.5 ± 14.0	ns
CI 95%	37.8-48.3	35.2-43.9		36.1-48.5	36.5-44.5	
MH	38.6 ± 13.9	39.8 ± 10.9	ns	38.8 ± 13.3	39.5 ± 11.1	ns
CI 95%	33.8-43.5	36.4-43.2		32.9-44.7	36.2-42.7	
SF-36v2	48.7 ± 9.5	42.1 ± 11.5	0.01	46.5 ± 10.4	44.1 ± 11.4	ns
PCS CI 95%	45.0-52.2	38.5-45.7		41.9-51.1	40.8-47.4	
SF-36v2	40.7 ± 12.8	40.6 ± 11.6	ns	40.5 ± 13.1	40.8 ± 11.7	ns
MCS CI 95%	35-9-45.5	37.0-44.4		34.6-46.3	37.4-44.2	
QLI						
HFSUB	20.2 ± 5.5	18.9 ± 4.7	ns	19.3 ± 5.9	19.5 ± 4.6	ns
CI 95%	18.2-22.3	17.5-20.4		16.7-22.0	18.2-20.8	
SOCSUB	22.5 ± 4.1	20.3 ± 3.7	0.01	21.5 ± 4.2	21.1 ± 3.9	ns
CI 95%	20.9-24.0	19.1-21.5		19.6-23.3	19.9-22.3	
PSPSUB	23.1 ± 4.9	21.6 ± 4.6	ns	22.7 ± 5.1	22.1 ± 4.6	ns
CI 95%	21.2-29.9	20.2-23.1		20.4-25.0	20.8-23.4	
FAMSUB	24.8 ± 4.6	24.5 ± 4.1	ns	24.8 ± 5.0	24.5 ± 3.9	ns
CI 95%	23.1-26.6	23.2-25.7		22.6-27.0	23.4-25.7	
QLI (Total)	21.9 ± 4.5	20.5 ± 3.8	ns	21.2 ± 4.8	21.1 ± 3.9	ns
CI 95%	20.2-23.6	19.3-21.7		19.1-23.3	19.9-22.2	

Data are presented as means ± SD and CI-confidence interval; the Mann–Whitney *U*-test was used for group comparisons (impaired vs. unimpaired). *P* values are expressed if they are <0.05. (AIS) Acceptance of Illness Scale, (HADS) Hospital Anxiety and Depression Scale, (BI) Barthel Index, SF-36v2 = (PF) physical functioning, (SF) social functioning, (RP) physical role functioning, (RE) emotional role functioning, (BP) bodily pain, (GH) general health, (VT) vitality, (MH) mental health, (PCS) physical component summary, (MSC) mental component summary, QLI-Quality of Life Index for Diabetes Version III = HFSUB- health and functioning subscale, SOCSUB- social and economic subscale, PSPSUB- psychological and spiritual subscale, FAMSUB- family subscale, *p* values are obtained by analyses using Mann–Whitney rank sum test.

**Table 3:** Functional assessment in the physical and psychological domains in relation to impaired versus unimpaired CV of the median nerve for both hands

	**Conduction velocities (mean = m/s)(CV)**					
	**Left hand**			**Right hand**		
	**Unimpaired**	**Impaired**	***P value***	**Unimpaired**	**Impaired**	***P value***
	**N = 48**	**N = 23**		**N = 41**	**N = 30**	
	**Mean = 54.5 ± 3.5 m/s**	**Mean = 46.1 ± 2.8 m/s**		**Mean = 54.1 ± 3.4 m/s**	**Mean = 45.8 ± 2.7 m/s**	
AIS	30.8 ± 7.9	28.8 ± 9.2	ns	30.6 ± 7.8	29.6 ± 9.1	ns
CI 95%	28.5-33.1	24.9-32.9		28.2-33.1	26.1-32.9	
HADS-D	4.8 ± 4.7	6.2 ± 4.2	ns	4.6 ± 4.4	6.1 ± 4.9	ns
CI 95%	3.4-6.2	4.4-8.1		3.3-6.0	4.3-8.0	
HADS-A	5.4 ± 5.1	6.2 ± 4.2	ns	5.6 ± 4.7	5.7 ± 5.1	ns
CI 95%	3.9-6.9	4.3-8.0		4.1-7.1	3.9-7.6	
BI	97.0 ± 8.5	94.3 ± 8.8	0.03	96.5 ± 9.0	95.6 ± 8.2	ns
CI 95%	94.5-99.5	90.5-98.1		93.6-99.3	92.6-98.7	
SF-36v2						
PF	45.8 ± 11.8	45.0 ± 12.5	ns	46.6 ± 10.9	44.1 ± 13.4	ns
CI 95%	42.4-49.3	39.6-50.4		43.2-50.1	39.1-49.1	
RP	41.6 ± 12.6	38.3 ± 14.3	ns	41.3 ± 13.2	39.6 ± 12.2	ns
CI 95%	37.9-45.3	32.2-44.6		37.1-45.5	34.6-44.5	
BP	51.9 ± 12.8	43.2 ± 14.6	0.03	49.6 ± 13.8	48.4 ± 14.1	ns
CI 95%	48.3-55.7	36.9-49.5		45.3-54.0	43.1-53.7	
GH	37.6 ± 12.3	30.9 ± 10.1	0.03	36.5 ± 11.7	33.8 ± 12.4	ns
CI 95%	33.9-41.1	26.5-35.3		32.9-40.2	29.2-38.5	
VT	45.9 ± 11.1	46.1 ± 11.7	ns	47.1 ± 9.9	44.5 ± 12.8	ns
CI 95%	42.7-49.1	41.1-51.2		43.9-50.2	39.7-49.3	
SF	44.8 ± 13.5	39.9 ± 14.6	ns	44.9 ± 12.8	41.0 ± 15.4	ns
CI 95%	40.9-48.8	33.6-46.4		40.9-49.1	35.2-46.8	
RE	42.8 ± 12.8	37.3 ± 15.6	ns	41.0 ± 14.4	41.1 ± 13.5	ns
CI 95%	39.1-46.6	30.6-44.0		36.5-45.5	36.1-46.1	
MH	40.7 ± 12.1	36.3 ± 10.3	ns	39.8 ± 12.4	38.6 ± 10.7	ns
CI 95%	37.2-44.3	31.9-40.8		35.9-43.8	34.5-42.6	
SF-36v2	46.1 ± 10.5	42.3 ± 12.0	ns	45.8 ± 10.5	43.4 ± 11.9	ns
PCS CI 95%	43.0-49.1	37.1-47.5		42.6-49.2	38.9-47.9	
SF-36v2	42.1 ± 11.7	37.7 ± 12.4	ns	41.3 ± 12.2	39.9 ± 12.1	ns
MCS CI 95%	38.7-45.6	32.3-43.1		37.4-45.1	35.4-44.4	
QLI						
HFSUB	20.4 ± 4.8	17.7 ± 5.3	0.05	19.9 ± 4.7	18.9 ± 5.6	ns
CI 95%	18.9-21.7	15.4-20.0		18.4-21.4	16.8-21.0	
SOCSUB	21.7 ± 4.1	20.1 ± 3.6	ns	21.6 ± 3.9	20.7 ± 4.1	ns
CI 95%	20.6-22.9	18.5-21.6		20.7-22.8	19.2-22.3	
PSPSUB	22.9 ± 5.0	21.0 ± 4.0	0.05	22.9 ± 4.8	21.4 ± 4.6	ns
CI 95%	21.5-24.4	19.3-22.7		21.4-24.5	19.7-23.1	
FAMSUB	24.8 ± 4.3	24.2 ± 4.3	ns	25.5 ± 4.0	23.4 ± 4.4	0.03
CI 95%	23.6-26.1	22.3-26.1		24.2-26.7	21.8-25.1	
QLI (Total)	21.7 ± 4.1	19.7 ± 4.1	0.05	21.6 ± 3.9	20.4 ± 4.3	ns
CI 95%	20.6-23.0	18.0-21.5		20.4-22.9	18.8-22.0	

Data are presented as means ± SD and CI-confidence interval; the Mann–Whitney *U*-test was used for group comparisons (impaired vs. unimpaired). *P* values are expressed if they are <0.05. (AIS) Acceptance of Illness Scale, (HADS) Hospital Anxiety and Depression Scale, (BI) Barthel Index, SF-36v2 = (PF) physical functioning, (SF) social functioning, (RP) physical role functioning, (RE) emotional role functioning, (BP) bodily pain, (GH) general health, (VT) vitality, (MH) mental health, (PCS) physical component summary, (MSC) mental component summary, QLI-Quality of Life Index for Diabetes Version III = HFSUB- health and functioning subscale, SOCSUB- social and economic subscale, PSPSUB- psychological and spiritual subscale, FAMSUB- family subscale, *p* values are obtained by analyses using Mann–Whitney rank sum test.

For the remaining analyses only CV are reported as these appeared to be the more sensitive of the electrophysiological measures of hand function used. Spearman rank correlations (r) were calculated for both left and right hand CV in relation to the parameters of interest measured in this study (see Table 4). CV for the left hand showed a strong negative correlation with HbA1c (r = −0.36; p = 0.004) and positive correlations with the BI (r = 0.31; p = 0.009) and the disease-specific measure of quality of life (QLI) (r = 0.24; p = 0.04).

**Table 4:** Spearman rank correlations (r) between left and right hand CV in relation to the parameters of interest measured

	**Conduction velocities (r):**	
	**Left hand**	**Right hand**
Age	−0.08	−0.14
Gender	0.15	0.08
Hb A1C	−0.36**	−0.15
Barthel Index	0.31**	0.07
AIS	0.15	0.07
HADS-D	−0.20	−0.11
HADS-A	−0.12	−0.01
SF-36v2 PCS	0.16	0.24*
SF-36v2 MCS	0.12	0.14
QLI	0.24*	0.20

*p < 0.05.

**p < 0.01.

On multiple regression analysis, the generic measure of quality of life (SF36) was significantly associated with depression (HADS-D), patient age, acceptance of illness (AIS), functional ability (BI) and conduction velocity for the right hand (CV-R) and these predictor variables accounted for 72.6% of the variance. In the model with disease-specific quality of life (QLI) as the dependent variable, there was a significant association with depression (HADS-D), the level of the c-fraction of glycosylated haemoglobin (HbA1c) and acceptance of illness (AIS), these predictor variables accounting for 63.1% of the variance.

## Discussion

It is generally accepted that the complications of diabetes are associated with poor metabolic control and duration of illness [32]. Hand abnormalities have been shown to be associated with duration of diabetes but not with metabolic control or other complications of the condition [17]. In the present study, hand dysfunctions as measured by electrophysiological recordings of distal motor latencies (DML) for the median nerve were associated with duration of illness only for the left hand, although a non-significant trend in a similar direction was observed also for the right hand and for both hands using nerve conduction velocities (CV). This result is in keeping with that of the previous study [17] and reflects the increased likelihood of peripheral neuropathy with longer duration, and therefore most probably, severity of illness. However, contrary to the results previously reported [17], there was also a tendency for longer distal latencies in the left hand to be associated with poor glycemic control, as reflected in the levels of HbA1c, and this reached significance for both hands on the measure of nerve conduction velocity (CV), used to determine median nerve function. Thus the results offer support for the view that hand function appears to be affected by poorer metabolic control in diabetes.

The aim of the present study was to investigate the effects of hand function on the quality of life of patients with diabetes. Hand impairments, as reflected in the electrophysiological measures used, occurred with equal frequencies in both hands. However, the results show that significant differences between impaired and unimpaired median nerve conductance groups occurred only for the left hand. Impaired groups achieved lower scores on the quality of life instruments, supporting the hypothesis that hand dysfunctions negatively affect quality of life. Specifically, the domains of quality of life (SF36v2) which distinguished between impaired and unimpaired groups were physical functioning (PF; p = .05), physical role performance (RP; p = .03), bodily pain (BP; p = .03) and general health (GH; p = .03) for the two electrophysiological measures used.

Earlier work concerning quality of life in patients with diabetes did not focus on any potential relationships with neurophysiological measurements [33-35], with the exception of Padua et al., who demonstrated an association between nerve conductance and quality of life in type 1 diabetes. They concluded that deficits in peripheral nerve function influence health related quality of life (SF-36v2) on the physical (PCS) but not the mental (MCS) components [15]. Our research provides additional support for these findings in demonstrating that deficits in median nerve conduction are associated with lower quality of life in the physical but not mental components of quality of life.

Of further interest are the results obtained with the QLI which is a disease-specific instrument for the measurement of quality of life in diabetes. Deficits in conductance of the left median nerve were found to reduce quality of life in relation to the SOCSUB- social and economic subscale, the HFSUB- health and functioning subscale and the PSPSUB- psychological and spiritual subscale, whilst conductance deficits in the right hand reduced quality of life in relation to the FAMSUB- family subscale. These data suggest that the QLI may be a useful instrument in the assessment of dimensions of quality of life, other than purely physical ones, in patients with diabetes and those with diabetic neuropathy. They provide preliminary support for the suggestion that the complications of diabetes may have broader implications for patients’ QOL than simply in the physical domain. Further studies are required to verify these results and to clarify which of the possible social, health, psychological and family aspects of functioning, if indeed any, are reliably affected. Future work should also focus on examining the relationships among key variables in order to determine the mechanisms behind the alleged effects on measures of outcome, such as QOL.

Differences were also found between impaired and unimpaired groups in relation to ADL as measured by the Barthel Index (BI) for both of the electrophysiological measures used. One previous study, [2] reporting that longer duration of diabetes was associated with an increased likelihood of diabetic neuropathy and difficulties in ADLs, also found that life satisfaction was high, and contrary to the findings of the present study, hand dysfunction did not have a significant association with the performance of ADLs. The reasons for this are not clear, but it may be that floor effects obscured the existence of any putative relationships i.e. high satisfaction with life may have been a reflection of few difficulties with ADLs.

Depression frequently coexists with chronic illnesses such as diabetes and significantly reduces the general health status and quality of life of the population examined [36,37]. Depression is associated with hyperglycemia in diabetes, which in turn, increases the risk of diabetic complications such as retinopathy, neuropathy and nephropathy [38,39]. There have been conflicting reports on the association between diabetic neuropathy and depression, but on balance, the evidence appears to support the existence of a relationship of this kind [40,41]. In our study, there were no differences between impaired and unimpaired groups on hand function in relation to the occurrence of anxiety or depression as measured by the HADS, possibly indicating that upper limb neuropathies are less likely to increase the occurrence of depression in diabetic patients or that the HADS is an insensitive measure of the psychopathology of diabetes. This latter conclusion may be unfounded however, as Lloyd et al., who consider the HADS to be an appropriate instrument for the clinical study of depression in adult patients with diabetes, have demonstrated a strong association between glycemic control and psychological symptoms, which was stronger for men than for women. They found a significant relationship between patient gender and glycaemic control (HbA1c) and with the occurrence of anxiety and depression [42]. These relationships require further clarification.

One of the consequences of lack of illness acceptance on the part of patients with diabetes is a failure to come to terms with the restrictions imposed by the illness, a lack of self-sufficiency, feelings of dependency on others and reduced self-efficacy[43,44]. Patients with diabetic neuropathy have greater difficulties in accepting their illness than patients with uncomplicated diabetes [43]. Our study however, did not reveal any significant differences between impaired and unimpaired groups in relation to electrophysiological recordings of hand function and levels of illness acceptance.

The main findings of this study indicate that hand function contributes to quality of life (SF36v2) in diabetes, although other factors, such as depression, patient age, acceptance of illness and functional performance are probably stronger determinants. The effect of hand dysfunction is relatively weak as the addition of this variable to the regression equation improves its ability to account for the variance by only a further 2%. With the measure of QLI, depression and acceptance of illness remain strong determinants of quality of life, but glycemic control (HbA1c) becomes an additional factor, although age and ADL are not. This supports the contention that the two measures of quality of life are likely to be tapping somewhat different domains of life satisfaction. Nevertheless mood and illness acceptance emerge as making the most significant contribution to quality of life. Further work needs to be done in order to understand the relationships between the variables associated with quality of life; for example, depression may be related to chronicity (and therefore age) in diabetes and may in turn, affect ADL performance and acceptance of illness, but other variations of these interrelationships among variables are possible.

A possible limitation of this study is that, in focusing on hand function, it does not provide a direct comparison with various control groups in order to test other determinants of quality of life in diabetes. One such comparison might involve patients with diabetes in whom peripheral neuropathies have been established for the lower limbs, and which might allow the differential effects of functional impairment in upper and lower limbs for QOL to be tested. However, no study is able to take account of all possibilities and future studies are needed to make the necessary comparisons. A complicating factor is that while peripheral neuropathies occur in the upper limbs, clinical symptoms are relatively rare, and when they do occur, the diabetic neuropathy is already well advanced in the legs and feet [45]. This means that dysfunctions of the lower limbs which impair mobility and are associated with diabetic morbidity may be more significant than hand function in terms of affecting quality of life, but it may be difficult to tease apart their differential effects. Nonetheless, this study, in having a specific focus, indicates that the effects of hand function, albeit small, make a significant contribution to quality of life.

As in all research attempting to disentangle the contribution of a variety of complex factors to clinical conditions, it may be the case that other factors that have not been accounted for in this study, also exert an influence on QOL in diabetes. One such factor may be the socio-economic status (SES) of people with diabetes; for example, Saydah & Lochner [46] have shown that in patients with lower SES, the risk of mortality is doubled compared to those of higher SES. It is plausible that SES may be associated with important aspects of illness management, such as conformity to dietary restrictions and medication regimens, those with poorer education levels and lower economic status having greater difficulty in adhering to advice in relation to the control of diabetes.

Systematic and reliable assessment of quality of life in patients with diabetes provides valuable information concerning those areas of functioning in which it is necessary to introduce significant changes. It is also of assistance in determining new methods of treatment and patient education [47]. The prevention of disability in patients with recognized diabetic neuropathy may be aided by rehabilitation procedures especially designed to improve hand function.

## Conclusions

This study has demonstrated a small, but significant effect of hand function on quality of life. The effect was found for the right hand on measures of CV, and may reflect the greater sensitivity of this measure and the fact that in people with right handed dominance (all of the sample studied), integrity of the right hand is especially important for both functional performance and quality of life. Indeed, patients in whom nerve conductance studies demonstrated impairment of the median nerve in the left hand were found to have difficulties with the activities of daily living as measured with the aid of the Barthel Index and a diminished quality of life in the area of physical functioning. Whilst no dependencies of this kind were found for the right hand, this may be because the neuropathic effects in the upper limbs are relatively small and function in the right hand has greater compensatory capacity than in the left.

The study has also demonstrated that depression and acceptance of illness are important contributors to quality of life and their interrelationship with other variables requires further study. Low mood and illness acceptance may be the consequences of diabetic comorbidity reflected in poor glycemic control and difficulty with ADLs or they may arise independently of these complications, affecting functional performance and quality of life.

As a result of the loss of functional ability in the hands, patients with diabetes may experience greater difficulties in manipulating objects and this may lead to an increased risk of accidents, for example, scalds and burns incurred as the result of ADLs. Another area of difficulty may be self-care activities, in particular those connected with the measurement of glycemic status and insulin injections. These findings may provide a basis for drawing greater attention to hand function in diabetes, both in terms of a better understanding of their contribution to functional capacity, but also to their potential effects on quality of life.

## Competing interests

The authors declare that they have no competing interest.

## Authors’ contributions

JL, BP, JK and WZ have contributed equally in the planning, data collection and analysis and interpretation of data, the writing process and discussion. MG, ZM, EK-K contributed to the analysis and interpretation of data, discussion and approval of the final revised version of the manuscript. All authors read and approved the final manuscript.
